# Combined with prognostic nutritional index and IgM for predicting the clinical outcomes of gastric cancer patients who received surgery

**DOI:** 10.3389/fonc.2023.1113428

**Published:** 2023-06-09

**Authors:** Zhongze Du, Hao Sun, Ruihu Zhao, Guiming Deng, Hongming Pan, Yanjiao Zuo, Rong Huang, Yingwei Xue, Hongjiang Song

**Affiliations:** Department of Gastrointestinal Surgery, Harbin Medical University Cancer Hospital, Harbin Medical University, Harbin, Heilongjiang, China

**Keywords:** prognostic nutritional index, IgM, gastric cancer, surgery, prognosis

## Abstract

**Objective:**

Although the survival rate of patients who undergo surgery for gastric cancer has greatly improved, still many patients have a poor prognosis. This retrospective study aimed to investigate the predictive ability of the PNI-IgM score, a combined prognostic nutritional index (PNI), and immunoglobulin M (IgM), on the prognosis of patients undergoing surgery for gastric cancer.

**Methods:**

340 patients with gastric cancer who underwent surgery from January 2016 to December 2017 were selected. The PNI-IgM score ranged from 1 to 3: score of 1, low PNI (< 48.45) and low IgM (< 0.87); score of 2, low PNI and high IgM, or high PNI and low IgM; score of 3, high PNI and high IgM. We compared the differences in disease-free survival (DFS) and overall survival (OS) among the three groups, while univariate and multivariate analyses calculated prognostic factors for DFS and OS. In addition, the nomograms were constructed based on the results of multivariate analysis to estimate the 1-, 3- and 5-year survival probability.

**Results:**

There were 67 cases in the PNI-IgM score 1 group, 160 cases in the PNI-IgM score 2 group, and 113 cases in the PNI-IgM score 3 group. The median survival times of DFS in the PNI-IgM score group 1, the PNI-IgM score group 2, and the PNI-IgM score group 3 were 62.20 months, not reached, and not reached, and 67.57 months vs. not reached vs. not reached in three groups for OS. Patients in the PNI-IgM score group 1 had a lower DFS than the PNI-IgM score group 2 (HR = 0.648, 95% CI: 0.418-1.006, *P* = 0.053) and the PNI-IgM score group 3 (HR = 0.337, 95% CI: 0.194-0.585, *P* < 0.001). In stratified analysis, PNI-IgM score 1 had a worse prognosis in the age < 60 years group and CA724 < 2.11 U/m group.

**Conclusion:**

PNI-IgM score is a novel combination of nutritional and immunological markers that can be used as a sensitive biological marker for patients with gastric cancer who undergo surgery. The lower the PNI-IgM score, the worse the prognosis.

## Introduction

According to the World Health Organization, gastric cancer was the fifth leading cancer in the world, with nearly 1.09 million new cases of gastric cancer worldwide in 2020. Gastric cancer is the fourth leading cause of cancer deaths, with almost 770,000 deaths worldwide in 2020 (1). In 2022, China is expected to have 509,000 new cases and 400,000 estimated deaths per year (2). Currently, radical gastrectomy is the preferred and primary treatment modality for patients with gastric cancer, while other treatments include endoscopic intervention followed by gastrectomy, adjuvant chemotherapy (ACT), or neoadjuvant chemotherapy (NACT) (3–5). In recent years, immunotherapy has also been applied to the treatment (6). Unfortunately, outcomes remain unsatisfactory as a significant number of patients develop local recurrence or distant metastases after resection (7). Therefore, it appears essential to identify potential biomarkers that can accurately select appropriate treatment strategies for patients and predict patient prognosis.

The nutritional status of patients with gastric cancer, which could predict the progression of the treated cancer, has been identified an important factor (8–11). It was common for patients with gastric cancer to suffer from malnutrition and cachexia due to reduced food intake and increased energy consumption (12). Cachexia was reported to affect approximately 50%-80% of cancer patients and was associated with 20%-40% of cancer deaths (13–15). Nutritional markers such as albumin (ALB), prealbumin (PALB), and body mass index (BMI) have been found to be independent prognostic factors for gastric cancer (16). The prognostic nutritional index (PNI), as a simple and readily available nutritional indicator, has been shown to be related to the prognosis of many malignancies, such as gastric cancer, lymphoma, pancreatic head cancer, head and neck tumors, etc. al (17–21). Additionally, the body’s Inflammatory response could also affect to tumor recurrence and metastasis. Some studies have reported that lymphocytes, neutrophils, and C-reactive protein (CRP) are associated with disease progression in patients with gastric cancer (22). Immunoglobulin M (IgM) was primarily responsible for the primary humoral immune response following initial antigen stimulation and has been shown to be closely associated with the prognosis of many malignancies (23–25). Taken together, the prognosis of gastric cancer patients with malnutrition and inflammation was worse.

A large number of previous researches have pointed out that composite indicators of nutrition and immunity, such as Glasgow Prognostic Score (GPS), platelet-to-lymphocyte (PLR), and Controlling Nutritional Status (CONUT) score could predict the prognosis of gastric cancer patients (26, 27). However, no study has investigated the validity of a mixed index of PNI and IgM (PNI-IgM score) to predict the prognosis of patients with gastric cancer who underwent surgery.

In this study, we evaluated the predictive effect of the PNI-IgM score on efficacy and prognosis in 340 patients with gastric cancer who underwent surgery. To further validate the PNI-IgM score, we performed a subgroup analysis and created nomograms.

## Materials and methods

### Patients

**Figure 1:**
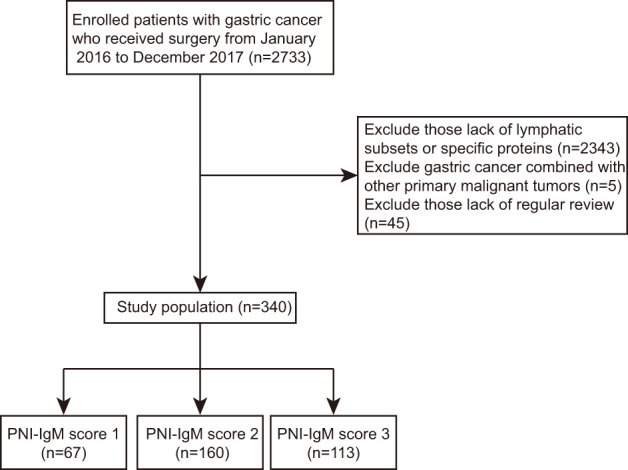
Flow chart of patients’ election in this study.

This is a retrospective study, so the Ethics Committee of Harbin Medical University Cancer Hospital waived informed consent. In total, we collected 340 consecutive patients with gastric cancer who received surgery at Harbin Medical University Cancer Hospital between January 2016 and December 2017 and were tested for lymphatic subsets and specific proteins. Statistical analysis of 340 patients and their clinical information was implemented according to the Helsinki Declaration and its amendments. All patients were included according to the following criteria: (1) all patients underwent surgical treatment; (2) all patients had no chronic disease; (3) all patients were tested for lymphatic subsets and specific proteins; (4) all patients did not display inflammatory response. (5) Patients with gastric cancer combined with other primary malignant tumors were excluded. Patients without complete clinical information and regular review after surgery were exclusion criteria. The flow chart of clinical case selection is shown in **Figure 1**. Electronic medical records system was used to collect clinical and pathological information.

### Data collection

Patients were followed up by telephone or outpatient visit, every 3-6 months during the first 2 years, and every 6-12 months from the 3rd to 5th year, and annually thereafter. Disease-free survival (DFS) was comprehended as the period from the first day of surgery date to the date of disease progression. The evidence of progression was obtained by chest and abdomen X-ray or computed tomography. DFS was also defined as the date of death, death from any cause, or the date of withdrawal from the follow-up. Overall survival (OS) was described as the period from the first day of surgery date to the date of death, the date of withdrawal from the follow-up, or the time of the last follow-up. Electronic medical record system was used to acquire patients’ clinical and pathological information.

The peripheral venous blood was collected in fasting state after admission in all patients. The counts of peripheral lymphocytes (L) were measured and analyzed by an automatic blood analyzer (BACKMAN COULTER LH750), the levels of peripheral albumin were measured and analyzed by an automatic blood analyzer (ADVIA-2400), and the levels of peripheral IgM were measured and analyzed by a specific protein analyzer (IMMAGE800). PNI was calculated as follows: PNI = albumin (g/L) + 5 × total lymphocyte counts (10^9^/L). The cut-off point was obtained by the receiver operating characteristic (ROC), which was based on OS for the prediction of patients’ death. The area under the ROC Curve (AUC) was used to evaluate the predictive ability of PNI and IgM. The optimal cut-off values of PNI and IgM with the highest Youden index were obtained.

### Statistical analysis

Continuous variables are presented as means with standard deviations or as medians with interquartile ranges. Categorical variables were expressed as percentages. The comparison between continuous variables used the t-tests, one-way ANOVA, Kruskal-Wails rank sum test. We used the Chi-square test or Fisher’s exact test to compare the discrepancies between categorical variables. The Kaplan-Meier survival curve was used to compute the survival rate and the Log-rank test to compare the survival time difference. Univariate and multivariate analyses were performed using the Cox proportional hazards model. Variables that achieved statistical significance at *P* < 0.05 were entered into the multivariate Cox regression analyses. Relative risks were assessed by the hazard ratio (HR) and 95% confidence interval (CI). The Cox proportional hazards regression model was constructed to analyze independent prognostic factors for DFS and OS. The nomograms were also constructed to predict the 1-, 3-, and 5-year survival probability for DFS and OS. The calibration curve analysis was used to assess the prognostic predictive ability of nomogram. All statistical analyses were completed through the R 4.1.3 (Vienna, Austria), IBM SPSS Statistics 25 (Chicago, IL, USA). Finally, we considered two-sided *P* values < 0.05 as statistical differences.

## Results

### Patient characteristics

The median age of patients was 60 years, and there were 105 women (30.9%) and 235 men (69.1%) in all two groups’ cases. The optimal cut-off value of PNI was 48.45. The optimal cut-off value of IgM was 0.87 g/L. According to the optimal cut-off values of PNI and IgM, all patients were divided into three groups: PNI-IgM score of 3 (n = 113): high IgM (≥ 0.87) and high PNI (≥ 48.45); PNI-IgM score of 2 (n = 160): high IgM (≥ 0.87) and low PNI (< 48.45), or low IgM (< 0.87) and high PNI (≥ 48.45); PNI-IgM score of 1(n = 67): low IgM (< 0.87) and low PNI (< 48.45). The Chi-square test or Fisher’s exact test showed that PNI-IgM score was related to melaena (*P* = 0.006), weight loss (*P* = 0.012), fatigue (*P* < 0.001), pTNM stage (*P* < 0.001) and tumor size (*P* = 0.003). The one-way ANOVA Kruskal-Wails rank sum test showed that PNI-IgM score was related to age (*P* < 0.001), BMI (*P* = 0.012), bleeding volume (*P* = 0.012). The detailed clinical characteristics of all 340 cases grouped by PNI-IgM score are displayed in **Table 1**.

**Table 1:** Clinical, pathological and laboratory information of all patients.

n	level	PNI-IgM Score 1	PNI-IgM Score 2	PNI-IgM Score 3	P
		67	160	113	
Sex	male	46 (68.7)	113 (70.6)	76 (67.3)	0.835
	female	21 (31.3)	47 (29.4)	37 (32.7)	
Age	median (interquartile range)	62.00 (53.00-68.00)	61.00 (54.00-67.00)	56.00 (48.00-62.00)	<0.001
Length of stay (d)	median (interquartile range)	15.00 (14.00-19.00)	17.00 (15.00-20.00)	17.00 (14.00-20.00)	0.052
BMI	Mean ± standard deviation	21.94 ± 3.68	22.54 ± 3.10	23.38 ± 3.17	0.012
ABO blood type	A	30 (44.8)	51 (31.9)	47 (41.6)	0.146
	B	19 (28.4)	47 (29.4)	28 (24.8)	
	O	16 (23.9)	45 (28.1)	23 (20.4)	
	AB	2 (3.0)	17 (10.6)	15 (13.3)	
Stomachache	no	18 (26.9)	53 (33.1)	28 (24.8)	0.295
	yes	49 (73.1)	107 (66.9)	85 (75.2)	
Melaena	no	48 (71.6)	116 (72.5)	99 (77.4)	0.006
	yes	19 (28.4)	44 (27.5)	14 (12.4)	
Weight loss	no	29 (43.3)	67 (41.9)	67 (59.3)	0.012
	yes	38 (56.7)	93 (58.1)	46 (40.7)	
Fatigue	no	28 (41.8)	98 (61.3)	87 (77.0)	<0.001
	yes	39 (58.2)	62 (38.8)	26 (23.0)	
Sour regurgitation	no	51 (76.1)	104 (65.0)	82 (72.6)	0.181
	yes	16 (23.9)	56 (35.0)	31 (27.4)	
pTNM	Tis/0	2 (3.0)	6 (3.8)	9 (8.0)	<0.001
	I	15 (22.4)	45 (28.1)	59 (52.2)	
	II	18 (26.9)	49 (30.6)	18 (15.9)	
	III	26 (38.8)	55 (34.4)	23 (20.4)	
	IV	6 (9.0)	5 (3.1)	4 (3.5)	
Radical resection	R0	62 (92.5)	152 (95.0)	107 (94.7)	0.753
	non-R0	5 (7.5)	8 (5.0)	6 (5.3)	
Type of surgery	distal gastrectomy	57 (85.1)	137 (85.6)	92 (81.4)	0.797
	proximal gastrectomy	1 (1.5)	4 (2.5)	4 (3.5)	
	total gastrectomy	4 (6.0)	11 (6.9)	9 (8.0)	
	exploratory laparotomy	2 (3.0)	2 (1.3)	1 (0.9)	
	gastroenterostomy	2 (3.0)	2 (1.3)	1 (0.9)	
	unknown	1 (1.5)	4 (2.5)	6 (5.3)	
Operation time(min)	median (interquartile range)	175.00 (150.00-200.00)	175.00 (150.00-210.00)	170.00 (145.00-198.00)	0.519
Bleeding volume(mL)	median (interquartile range)	100.00 (100.00-200.00)	100.00 (100.00-200.00)	100.00 (50.00-175.00)	0.012
Tumor site	upper 1/3	3 (4.5)	4 (2.5)	5 (4.4)	0.247
	middle 1/3	5 (7.5)	21 (13.1)	18 (15.9)	
	low 1/3	47 (70.1)	118 (73.8)	82 (72.3)	
	whole	12 (17.9)	17 (10.6)	8 (7.1)	
Tumor size	<50 mm	27 (40.3)	73 (45.6)	73 (64.6)	0.003
	≥50 mm	36 (53.7)	83 (51.9)	35 (31.0)	
	unknown	4 (6.0)	4 (2.5)	5 (4.4)	
Differentiation	poorly differentiated	20 (29.9)	59 (36.9)	48 (42.5)	0.245
	moderately differentiated	39 (58.2)	83 (51.9)	45 (39.8)	
	well differentiated	4 (6.0)	11 (6.9)	13 (11.5)	
	unknown	4 (6.0)	7 (4.4)	7 (6.2)	
Lauren type	intestinal	34 (50.7)	73 (45.6)	54 (47.8)	0.877
	diffuse	9 (13.4)	30 (18.8)	21 (18.6)	
	mixed	21 (31.3)	51 (31.9)	31 (27.4)	
	unknown	3 (4.5)	6 (3.8)	7 (6.2)	
ALT (U/L)	median (interquartile range)	16.00 (12.40-20.00)	18.00 (13.13-25.00)	20.00 (14.00-25.85)	0.017
AST (U/L)	median (interquartile range)	19.00 (15.00-23.00)	20.00 (17.00-25.75)	21.00 (17.00-26.00)	0.120
γ- GT (U/L)	median (interquartile range)	13.00 (10.00-24.00)	16.00 (11.00-24.00)	21.00 (14.00-32.00)	<0.001
LDH (U/L)	median (interquartile range)	158.00 (140.00-175.00)	159.00 (141.25-180.00)	163.00 (147.00-183.00)	0.716
TBIL (μmol/L)	median (interquartile range)	9.32 (6.60-13.38)	11.23 (8.55-15.36)	12.20 (9.39-15.71)	0.001
TP (g/L)	median (interquartile range)	61.10 (58.00-64.00)	67.50 (64.00-70.90)	71.50 (68.00-74.50)	<0.001
ALB (g/L)	median (interquartile range)	36.90 (34.60-38.00)	41.00 (39.00-43.00)	43.00 (41.00-45.00)	<0.001
GLOB (g/L)	median (interquartile range)	24.30 (22.00-27.00)	26.00 (24.00-28.00)	28.00 (26.00-31.00)	<0.001
Urea (mmol/L)	median (interquartile range)	5.70 (4.80-7.20)	5.70 (4.60-6.90)	5.50 (4.60-6.70)	0.805
CREA (μmol/L)	median (interquartile range)	78.00 (65.00-91.00)	82.50 (74.00-92.00)	83.00 (76.00-92.00)	0.092
UA (μmol/L)	median (interquartile range)	271.00 (226.00-323.00)	300.00 (241.25-354.75)	310.00 (264.50-364.00)	0.002
ALP (U/L)	median (interquartile range)	66.00 (58.00-84.00)	72.50 (61.00-85.00)	77.00 (63.00-93.50)	0.027
WBC (10^9^/L)	median (interquartile range)	5.63 (4.71-6.69)	6.48 (5.31-8.03)	6.67 (5.49-7.62)	<0.001
Mono (10^9^/L)	median (interquartile range)	0.44 (0.31-0.53)	0.47 (0.36-0.67)	0.48 (0.39-0.58)	0.081
Eosi (10^9^/L)	median (interquartile range)	0.11 (0.07-0.19)	0.13 (0.06-0.22)	0.14 (0.07-0.22)	0.431
Baso (10^9^/L)	median (interquartile range)	0.02 (0.02-0.04)	0.02 (0.02-0.04)	0.03 (0.02-0.04)	0.632
RBC (10^12^/L)	Mean ± standard deviation	4.10 ± 0.60	4.42 ± 0.60	4.68 ± 0.46	<0.001
P (10^9^/L)	median (interquartile range)	248.00 (192.00-323.00)	247.50 (202.50-297.00)	235.00 (203.50-179.50)	0.611
CEA (10^9^/L)	median (interquartile range)	1.87 (1.05-4.47)	2.00 (1.34-3.57)	1.94 (1.16-2.78)	0.644
CA199 (U/mL)	median (interquartile range)	11.45 (6.03-11.45)	10.21 (6.04-16.72)	9.81 (6.31-16.01)	0.292
CA724 (U/mL)	median (interquartile range)	3.01 (1.35-9.80)	1.90 (1.18-4.66)	2.13 (1.01-5.33)	0.260
CA125II (U/mL)	median (interquartile range)	10.62 (6.94-17.34)	10.12 (7.42-13.74)	10.26 (7.75-14.15)	0.547
IgA (g/L)	median (interquartile range)	2.08 (1.50-2.79)	2.11 (1.54-2.79)	2.61 (1.88-3.10)	<0.001
IgG (g/L)	median (interquartile range)	9.19 (7.76-11.10)	9.95 (8.51-11.76)	11.50 (9.73-12.85)	<0.001
TRF (g/L)	median (interquartile range)	2.19 (1.92-2.57)	2.10 (1.82-2.50)	2.22 (2.02-2.62)	0.111
KAP (g/L)	median (interquartile range)	7.19 (5.83-8.36)	8.18 (6.99-9.44)	9.40 (7.97-11.10)	<0.001
LAM (g/L)	median (interquartile range)	4.34 (3.40-5.39)	4.69 (4.02-5.47)	5.22 (4.48-6.07)	<0.001
K/L	median (interquartile range)	1.75 (1.47-1.94)	1.77 (1.54-1.97)	1.81 (1.57-2.06)	0.298
CD3 + (%)	median (interquartile range)	72.20 (64.70-76.70)	69.40 (62.13-74.98)	69.40 (65.25-79.20)	0.423
CD3 +/CD4 + (%)	median (interquartile range)	41.90 (39.40-48.10)	40.60 (34.93-46.70)	41.00 (34.85-45.85)	0.141
CD3 +/CD8 + (%)	median (interquartile range)	21.70 (16.40-29.20)	21.90 (16.80-28.35)	23.30 (17.85-28.35)	0.658
CD4 +/CD8 +	median (interquartile range)	1.92 (1.34-2.79)	1.91 (1.33-2.52)	1.77 (1.31-2.29)	0.577
CD3 +/CD4 + CD8 + (%)	median (interquartile range)	0.20 (0.10-0.40)	0.30 (0.10-0.50)	0.30 (0.20-0.70)	0.004
CD19 + (%)	median (interquartile range)	10.60 (7.90-14.00)	10.20 (8.00-13.88)	11.20 (8.45-14.90)	0.253
CD3 -/CD16 + CD56 + (%)	median (interquartile range)	13.40 (9.30-21.00)	15.70 (10.08-22.63)	14.90 (9.20-20.45)	0.473
CD3+/CD16+ CD56 + (%)	median (interquartile range)	2.10 (1.10-3.90)	1.65 (0.90-3.00)	1.80 (1.05-4.25)	0.330

In this study, we also collected patients’ nutritional and hematological parameters before surgery, including total protein (TP), PALB, alanine aminotransferase (ALT), aspartate transaminase (AST), glutamyl transpeptidase (γ- GT), lactate dehydrogenase (LDH), total bilirubin (TBIL), globulin (GLOB), urea, creatinine (CREA), urate (UA), alkaline phosphatase (ALP), monocyte (Mono), eosinophils (Eosi), basophil (Baso), red blood cell (RBC), platelet (P), carcinoembryonic antigen (CEA), carbohydrate antigen 199 (CA199), carbohydrate antigen 724 (CA724), carbohydrate antigen 125II (CA125II). We also collected lymphatic subsets and specific proteins, including immunoglobulin A (IgA), immunoglobulin G (IgG), transferrin (TRF), light-chain immunoglobulin (KAP), heavy-chain immunoglobulin (LAM), KAP/LAM, CD3 + cells (T cells), CD3 +/CD4 + cells (Th cells), CD3 +/CD8 + cells (CTL cells), CD4 +/CD8+ cells, CD3 +/CD4 + CD8 + cells, CD19 + cells (B cells), CD3 -/CD16 + CD56 + cells (NK cells), CD3 +/CD16 + CD56 + cells (NKT cells). We analyzed their relationship to the PNI-IgM score by Kruskal-Wails rank sum test (**Table 1**). We found that the PNI-IgM score was related to ALT (*P* = 0.017), γ- GT (*P* < 0.001), TBIL (*P* < 0.001), TP (*P* < 0.001), ALB (*P* < 0.001), GLOB (*P* < 0.001), UA (*P* = 0.002), ALP (*P* = 0.027), WBC (*P* < 0.001), RBC (*P* < 0.001), IgA (*P* < 0.001), IgG (*P* < 0.001), KAP (*P* < 0.001), LAM (*P* < 0.001) and CD3 +/CD4 + CD8 + cells (*P* = 0.004).

### Univariate and multivariate Cox hazard analysis for DFS and OS

According to univariate analysis, the prognosis factors for patients’ DFS in this study were age (*P* = 0.001), BMI (*P* = 0.043), tumor size (*P* < 0.001), pTNM stage (*P* < 0.001), radical resection (*P* < 0.001), TBIL (*P* = 0.020), Eosi (*P* = 0.032), RBC (*P* = 0.032), CA724 (*P* < 0.001), CA125II (*P* = 0.003), IgG (*P* = 0.028), PNI-IgM score (*P* < 0.05). And the prognosis factors of patients in this study for OS were age (*P* = 0.001), melaena (*P* = 0.048), tumor size (*P* < 0.001), pTNM stage (*P* < 0.001), radical resection (*P* < 0.001), TBIL (*P* = 0.016), Eosi (*P* = 0.030), RBC (*P* = 0.031), CA724 (*P* = 0.004), CA125II (*P* = 0.004), IgG (*P* = 0.036), PNI-IgM score (*P* < 0.05). The multivariate analysis indicated that age (*P* = 0.036 vs. *P* = 0.035), pTNM stage (*P* < 0.001 vs. *P* < 0.001), radical resection (*P* = 0.006 vs. *P* = 0.001), and CA724 (*P* = 0.027 vs. *P* = 0.015) were both independent prognostic factors for DFS and OS. In addition, melaena (*P* = 0.003) was the independent prognostic factor for OS (**Table 2**).

**Table 2:** Univariate and multivariate analysis for DFS and OS.

Parameters	DFS				OS			
	Univariate analysis	P value	Multivariate analysis	P value	Univariate analysis	P value	Multivariate analysis	P value
	Hazard ratio (95%CI)		Hazard ratio (95%CI)		Hazard ratio (95%CI)		Hazard ratio (95%CI)	
Sex (Male vs. Female)	0.900(0.590-1.374)	0.627			0.886(0.581-1.352)	0.575		
Age (<60 vs. ≥60)	1.982(1.326-2.963)	0.001	1.603(1.032-2.490)	0.036	2.005(1.341-2.997)	0.001	1.613(1.035-2.515)	0.035
Length of Stay (<16.5 d vs. ≥16.5 d)	0.849(0.579-0.244)	0.401			0.867(0.592-1.271)	0.464		
BMI (22.84 kg/m^2^ vs. ≥22.84 kg/m^2^)	0.672(0.458-0.988)	0.043	0.865(0.573-1.306)	0.490	0.687(0.468-1.009)	0.056	0.925(0.613-1.396)	0.710
Stomachache (No vs. Yes)	1.353(0.869-2.107)	0.181			1.371(0.881-2.135)	0.163		
Melaena (No vs. Yes)	1.507(0.991-2.290)	0.055			1.525(1.004-2.318)	0.048	1.967(1.260-3.070)	0.003
Fatigue (No vs. Yes)	1.395(0.950-2.049)	0.090			1.425(0.970-2.093)	0.071		
Sour regurgitation (No vs. Yes)	0.220(0.814-1.830)	0.335			1.195(0.797-1.792)	0.388		
Tumor size (<50 mm vs. ≥50 mm + unkonwn)	3.296(2.167-5.014)	<0.001	1.393(0.869-2.234)	0.169	3.311(2.177-5.037)	<0.001	1.491(0.938-2.370)	0.091
pTNM (0/Tis + I + II vs. III + IV)	6.807(4.492-10.314)	<0.001	4.662(2.923-7.436)	<0.001	6.278(4.151-9.496)	<0.001	4.466(2.805-7.109)	<0.001
Radical resection (R0 vs. Non-R0)	2.014(1.505-2.694)	<0.001	1.551(1.132-2.125)	0.006	2.065(1.543-2.763)	<0.001	1.699(1.241-2.325)	0.001
Operation time (<175 min vs. ≥175 min)	1.018(0.696-1.491)	0.926			1.026(0.701-1.503)	0.894		
Bleeding volume (<100 ml vs. ≥100 ml)	1.629(0.969-2.737)	0.065			1.657(0.986-2.785)	0.056		
ALT (<18 U/L vs. ≥18 U/L)	0.900(0.615-1.317)	0.588			0.884(0.604-1.294)	0.526		
AST (<20 U/L vs. ≥20 U/L)	0.895(0.611-1.311)	0.569			0.891(0.608-1.307)	0.556		
γ- GT (<17 U/L vs. ≥17 U/L)	0.943(0.644-1.380)	0.762			0.981(0.670-1.436)	0.921		
LDH (<159.5 U/L vs. ≥159.5 U/L)	1.101(0.752-1.611)	0.622			1.113(0.760-1.630)	0.582		
TBIL (<10.77 μmol/L vs. ≥10.77 μmol/L)	0.632(0.430-0.931)	0.020	0.810(0.533-1.231)	0.324	0.622(0.422-0.915)	0.016	0.709(0.464-1.083)	0.111
TP (<68 g/L vs. ≥68 g/L)	0.833(0.569-1.220)	0.349			0.845(0.577-1.238)	0.388		
ALB (<41 g/L vs. ≥41 g/L)	0.724(0.494-1.060)	0.097			0.716(0.489-1.050)	0.087		
GLOB (<26.9 g/L vs. ≥26.9 g/L)	0.899(0.614-1.317)	0.586			0.917(0.626-1.344)	0.658		
A/G (<1.5 vs. ≥1.5)	0.780(0.529-1.150)	0.210			0.767(0.520-1.132)	0.182		
Urea (<5.65 mmol/L vs. ≥5.65 mmol/L)	0.970(0.663-1.420)	0.877			0.882(0.678-1.452)	0.967		
CREA (<82 μmol/L vs. ≥ 82 μmol/L)	1.149(0.783-1.686)	0.478			1.154(0.786-1.693)	0.465		
UA (<300 μmol/L vs. ≥300 μmol/L)	0.923(0.631-1.351)	0.679			0.926(0.633-1.355)	0.693		
ALP (<73 U/L vs. ≥73 U/L)	1.090(0.744-1.595)	0.660			1.124(0.768-1.646)	0.547		
MoNo (<0.47 10^9^/L vs. ≥0.47 10^9^/L)	0.994(0.679-1.455)	0.973			1.029(0.703-1.507)	0.883		
Eosi (<0.13 10^9^/L vs. ≥0.13 10^9^/L)	0.657(0.447-0.965)	0.032	0.886(0.588-1.334)	0.561	0.653(0.444-0.960)	0.030	0.872(0.578-1.315)	0.512
Baso (<0.02 10^9^/L vs. ≥0.02 10^9^/L)	0.958(0.612-1.500)	0.851			0.921(0.588-1.442)	0.718		
RBC (<4.47 10^12^/L vs. ≥4.47 10^12^/L)	0.655(0.445-0.963)	0.032	1.044(0.670-1.627)	0.848	0.653(0.443-0.961)	0.031	1.088(0.703-1.685)	0.705
P (<247 10^9^/L vs. ≥247 10^9^/L)	1.012(0.752-1.613)	0.619			1.110(0.758-1.625)	0.593		
CEA (<1.98 ng/mL vs. ≥1.98 ng/mL)	1.419(0.966-2.083)	0.075			1.467(0.999-2.154)	0.051		
CA199 (<10.13 U/mL vs. ≥10.13 U/mL)	1.369(0.933-2.009)	0.108			1.373(0.935-2.015)	0.106		
CA724 (<2.11 U/mL vs. ≥2.11 U/mL)	2.169(1.454-3.234)	<0.001	1.656(1.044-2.590)	0.027	2.123(1.423-3.165)	<0.001	1.749(1.117-2.737)	0.015
CA125II (<10.22 U/mL vs. ≥10.22 U/mL)	1.796(1.217-2.652)	0.003	1.262(0.827-1.925)	0.281	1.771(1.199-2.614)	0.004	1.261(0.823-1.934)	0.287
IgA (<2.25 g/L vs. ≥2.25 g/L)	0.796(0.543-1.166)	0.242			0.804(0.549-1.178)	0.263		
IgG (<10.15 g/L vs. ≥10.15 g/L)	0.648(0.439-0.954)	0.028	1.104(0.722-1.687)	0.648	0.660(0.488-0.973)	0.036	1.150(0.749-1.765)	0.523
TRF (<2.17 g/L vs. ≥2.17 g/L)	0.847(0.578-1.240)	0.393			0.869(0.594-1.273)	0.472		
KAP (<8.3 g/L vs. ≥8.3 g/L)	0.892(0.609-1.307)	0.599			0.918(0.627-1.344)	0.659		
LAM (<4.75 g/L vs. ≥4.75 g/L)	0.955(0.652-1.398)	0.813			0.959(0.655-1.404)	0.831		
K/L (<1.78 vs. ≥1.78)	0.863(0.590-1.264)	0.450			0.904(0.617-1.323)	0.602		
CD3 + (<69.5% vs. ≥69.5%)	1.328(0.904-1.950)	0.148			1.342(0.914-1.972)	0.133		
CD3 +/CD4 + (<41.15% vs. ≥41.15%)	0.908(0.620-1.329)	0.620			0.916(0.625-1.340)	0.650		
CD3 +/CD8 + (<22.3% vs. ≥22.3%)	1.234(0.841-1.810)	0.283			1.232(0.840-1.808)	0.285		
CD4 +/CD8 + (<1.87 vs. ≥1.87)	0.906(0.619-1.328)	0.612			0.904(0.617-1.323)	0.602		
CD3 +/CD4+ CD8+ (<0.3% vs. ≥0.3%)	1.008(0.68-1.476)	0.966			1.039(0.710-1.521)	0.844		
CD19 + (<10.7% vs. ≥10.7%)	0.781(0.533-1.145)	0.206			0.766(0.523-1.123)	0.172		
CD3 -/CD16 + CD56 + (<14.85% vs. ≥14.85%)	0.715(0.487-1.050)	0.087			0.722(0.491-1.060)	0.097		
CD3 +/CD16 + CD56 + (1.8% vs. ≥1.8%)	1.278(0.871-1.876)	0.209			1.314(0.896-1.928)	0.162		
PNI-IgM Score 1	Ref		Ref		Ref		Ref	
PNI-IgM Score 2	0.648(0.418–1.006)	0.053	1.003(0.624-1.610)	0.991	0.631(0.407-0.978)	0.040	0.904(0.564-1.450)	0.675
PNI-IgM Score 3	0.337(0.194-0.585)	<0.001	0.722(0.391-1.333)	0.298	0.327(0.188-0.568)	<0.001	0.701(0.379-1.297)	0.258

### Stratified analyses by potential effect modifiers

**Figure 2:**
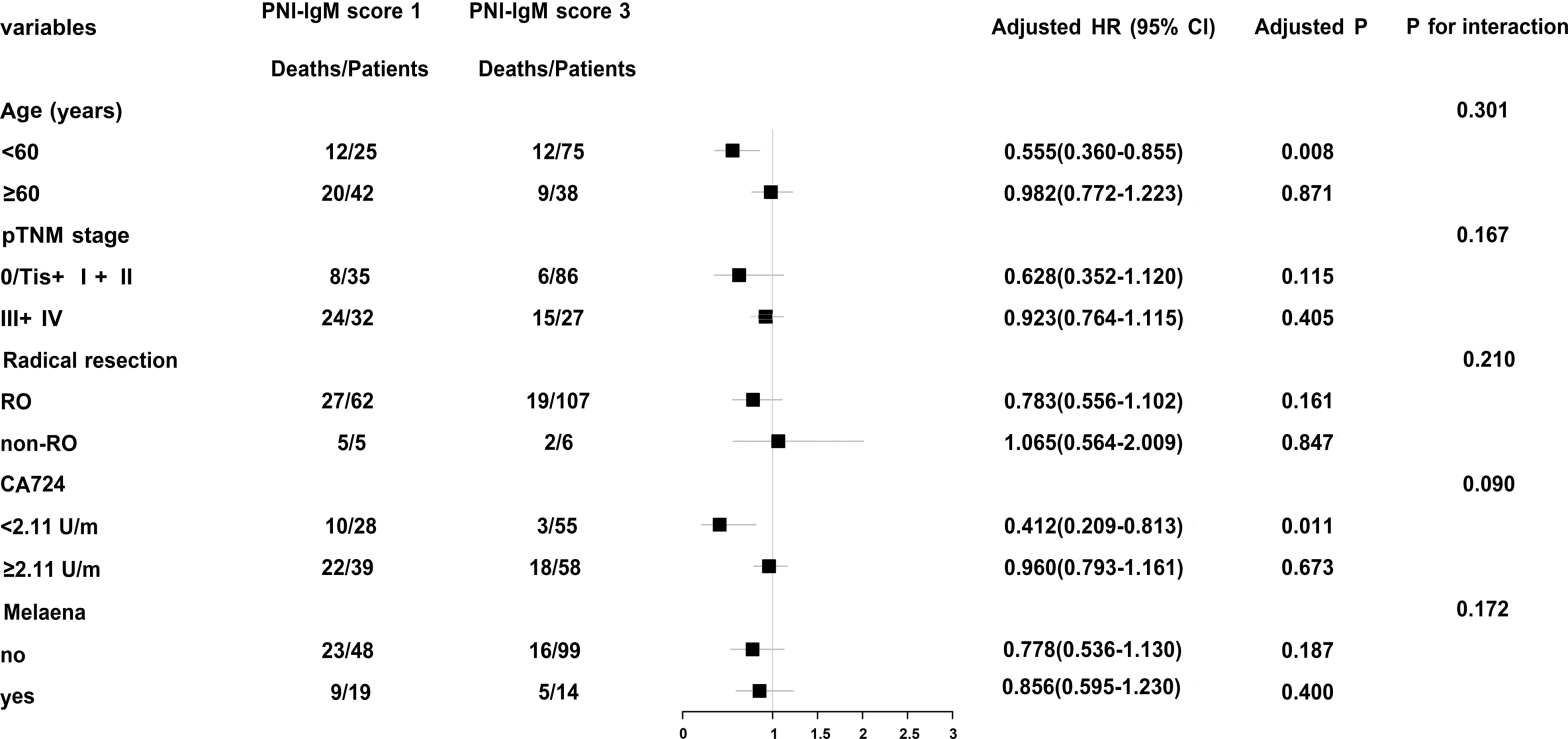
The stratificationan alysis of PNI-IgM score for OS.

In order to further study PNI-IgM score, we conducted a stratified analysis and found that patients with PNI-IgM score 1 had shorter OS in age < 60 years group (HR = 0.555, 95% CI: 0.360-0.855, *P* = 0.008) and in CA724 < 2.11U/m group (HR = 0.412, 95% CI: 0.209-0.813, *P* = 0.011) (**Figure 2**).

### PNI-IgM score and prognosis

**Figure 3:**
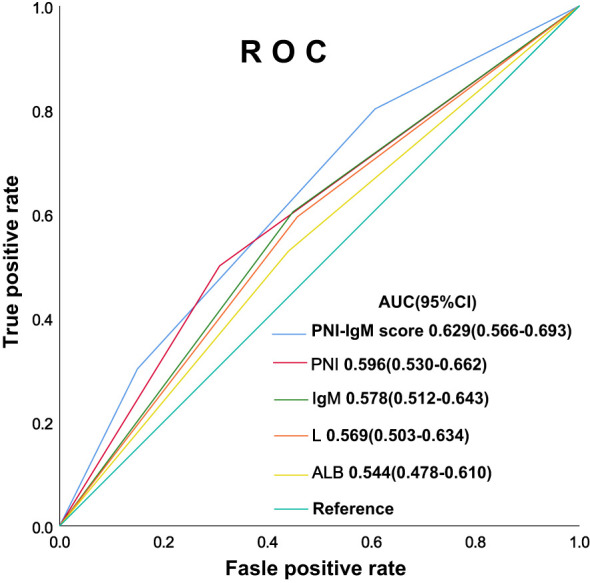
The ROC curves of PNI-IgM score, PNI, IgM, ALB, L.

**Figure 4:**
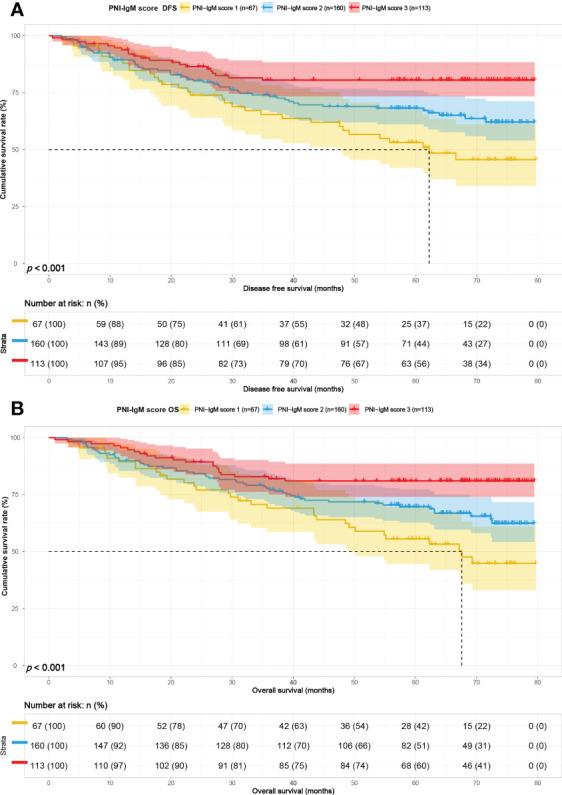
PNI-IgM score related survival curve of **(A)** DFS and **(B)** OS in all patients.

The PNI-IgM score 1 group’s median survival time for DFS was 62.20 months, the 1-, 3-, and 5-year DFS probability was 89.4% (95% CI: 82.3%-97.1%), 65.4% (95% CI: 54.7%-78.3%), 53.1% (95% CI: 41.9%-67.3%). The PNI-IgM score 1 group’s median survival time for OS was 67.57 months, the 1-, 3-, and 5-year OS probability was 89.5% (95% CI: 82.4%-97.2%), 69.0% (95% CI: 58.6%-81.3%), 55.6% (95% CI: 44.5%-69.4%). The PNI-IgM score 2 group’s median survival time for DFS and OS were both not achieved. The 1-, 3-, and 5-year DFS and OS probability were 89.3% (95% CI: 84.6%-94.2%), 74.0% (95% CI: 67.4%-81.3%), 68.2% (95% CI: 61.1%-76.1%); 89.9% (95% CI: 85.4%-94.7%), 77.8% (95% CI: 71.6%-84.6%), 69.7% (95% CI: 62.8%-77.3%), respectively. The PNI-IgM score 3 group’s median survival time for DFS and OS were both not achieved. The 1-, 3-, and 5-year DFS and OS probability were 93.8% (95% CI: 89.4%-98.4%), 80.5% (95% CI: 73.4%-88.4%), 80.5% (95% CI: 73.4%-88.4%); 95.6% (95% CI: 91.9%-99.4%), 82.0% (95% CI: 75.1%-89.4%), 81.0% (95% CI: 74.0%-88.7%), respectively. To further determine whether the PNI-IgM score could predict the prognosis of gastric cancer patients. The ROC curves were based on OS for the prediction of patients’ death. For the traditional clinicopathologic factors, including ALB, L, PNI, and IgM, each feature and the combined PNI-IgM score were plotted, and the point with the highest AUC was illustrated on the ROC curve. The PNI-IgM score exhibited a higher prognostic accuracy for DFS and OS than PNI and other clinicopathological risk factors (**Figure 3**). Patients with the PNI-IgM score 1 have a worse DFS than patients with the PNI-IgM score 2 (HR = 0.648, 95% CI: 0.418-1.006, *P* = 0.053) or the PNI-IgM score 3 (HR = 0.337, 95% CI: 0.194-0.585, *P* < 0.001). Patients with the PNI-IgM score 1 have a shorter OS than patients with the PNI-IgM score 2 (HR = 0.631, 95% CI: 0.407-0.978, *P* = 0.040) or the PNI-IgM score 3 (HR = 0.327, 95% CI: 0.188-0.568, *P* < 0.001) (**Figures 4A, B**).

### Survival for pTNM stage

**Figure 5:**
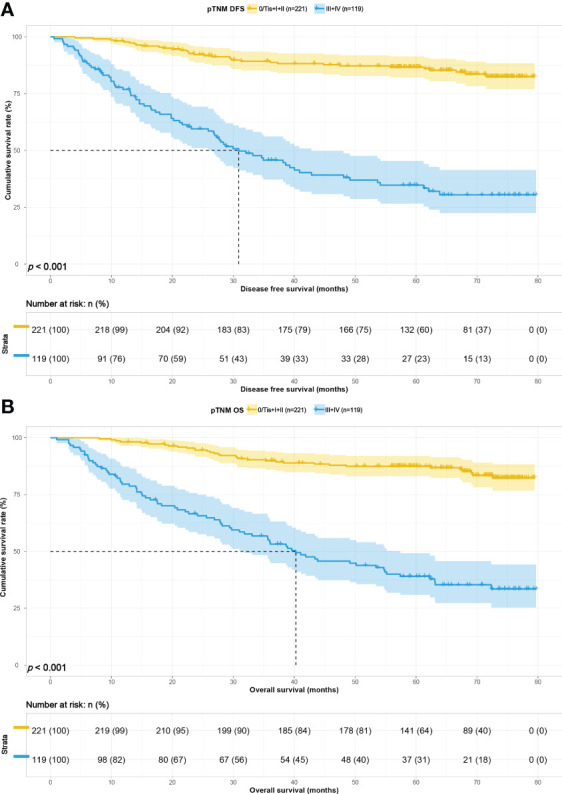
pTNM related survival curve of **(A)** DFS and **(B)** OS in all patients.

To study the predictive ability of PNI-IgM score for prognosis of gastric cancer patients in correlation with pTNM stage, we divided the 340 patients into early pTNM stage (0/Tis + I + II) group (221 patients) and advanced pTNM stage (III + IV) group (119 patients). The median survival time for DFS and OS in the early pTNM stage group were both not reached. The 1-, 3-, and 5-year DFS and OS probability were 98.2% (95% CI: 96.4%-100.0%) vs. 98.2% (95% CI: 96.4%-100.0%), 89.2% (95% CI: 85.2%-93.5%) vs. 89.9% (95% CI: 86.0%-94.0%), 86.7% (95% CI: 82.2%-91.4%) vs. 87.4% (95% CI: 83.1%-92.0%). The median survival time for DFS and OS in the advanced pTNM stage group were 30.90 months and 40.27 months. The 1-, 3-, and 5-year DFS and OS probability were 76.9% (95% CI: 69.6%-84.9%) vs. 79.6% (95% CI: 72.7%-87.2%); 45.7% (95% CI: 37.2%-56.2%) vs. 54.1% (95% CI: 45.7%-64.0%); 34.7% (95% CI: 26.6%-45.4%) vs. 39.0% (95% CI: 30.9%-49.3%). Patients with the PNI-IgM score 1 have a shorter DFS than patients with the PNI-IgM score 2 (HR = 0.814, 95% CI: 0.356-1.860, *P* = 0.626) or the PNI-IgM score 3 (HR = 0.278, 95% CI: 0.096-0.800, *P* = 0.018). Patients with the PNI-IgM score 1 also have a shorter OS than patients with the PNI-IgM score 2 group (HR = 0.821, 95% CI: 0.359-1.876, *P* = 0.640) or the PNI-IgM score 3 group (HR = 0.280, 95% CI: 0.097-0.808, *P* = 0.019). Patients in the advanced pTNM stage had lower DFS (HR = 4.394, 95% CI: 2.737-7.055, *P* < 0.001) and OS (HR = 4.466, 95% CI: 2.805-7.109, *P* < 0.001) than those early pTNM stage patients (**Figures 5A, B**).

### Construction of nomograms to predict DFS and OS

**Figure 6:**
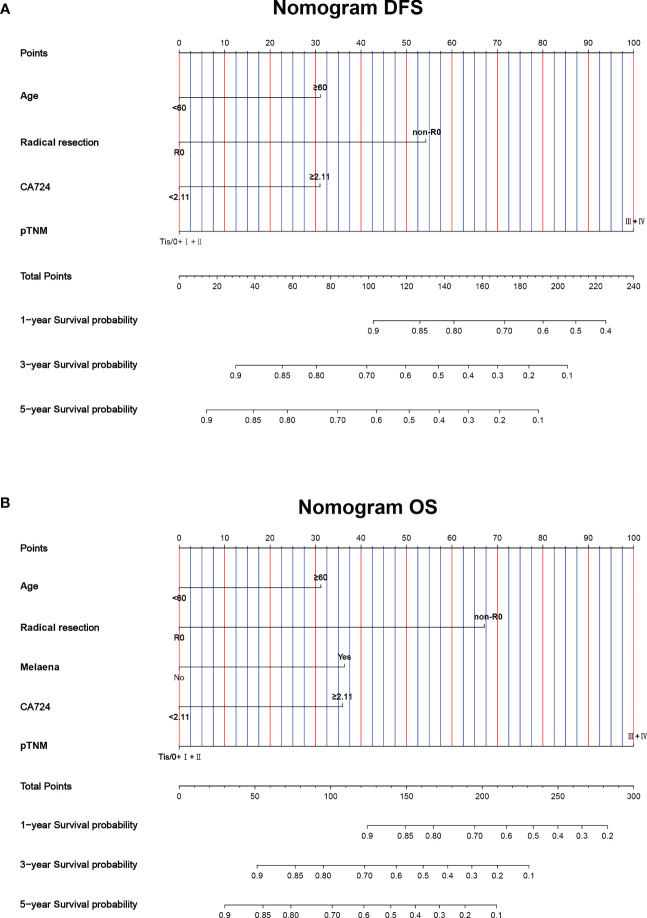
Nomogram for predicting 1-, 3-, 5-year survival probability of **(A)** DFS and **(B)** OS.

**Figure 7:**
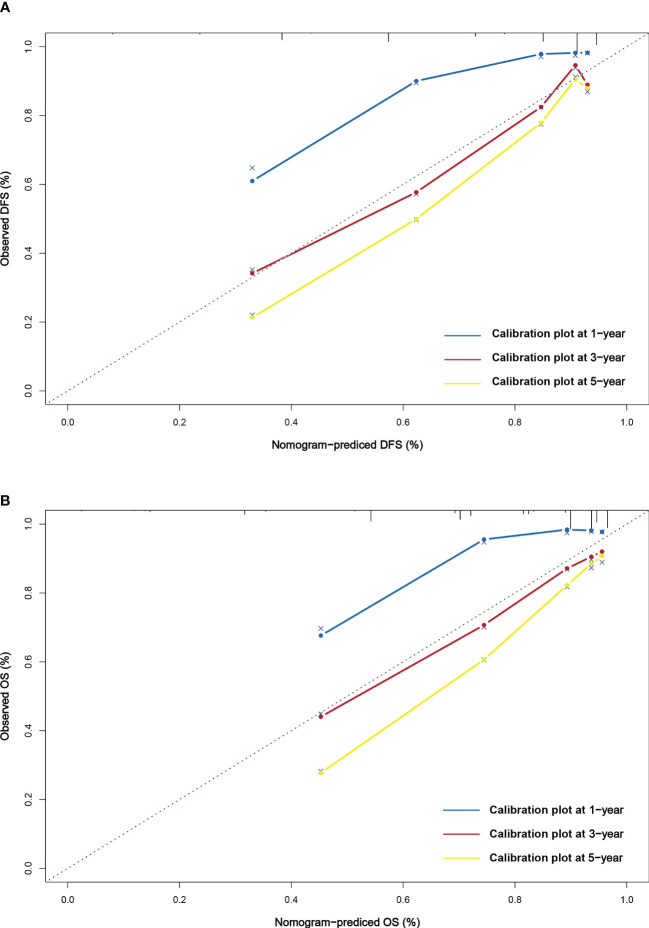
Calibration curves for predicting DFS **(A)** and OS **(B)** at 1-,3-, and 5-years.

This study found that age, radical resection, CA724, and pTNM stage were the independent prognostic factors for DFS. By constructing Cox proportional hazard regression model, age, melaena, radical resection, CA724, and pTNM stage were the independent prognostic factors for OS. Based on the results of multivariate analysis, the nomograms to predict the 1-, 3-, and 5-year survival probability for DFS and OS were established (**Figures 6A, B**). The C-index and 95% CI for predicting the survival probability of DFS and OS were 0.770 (0.724-0.817) and 0.772 (0.724-0.819). Calibration curves for the DFS probability at 3-, 5-years in the demonstrated satisfactory consistency between the nomogram-predicted and actual survival. The calibration curves for the probability of OS at 3-, 5-years also suggested correlation between the observed and nomogram-predicted survival (**Figures 7A, B**).

## Discussion

Gastric cancer is a common malignant tumor in China, with the third highest incidence and mortality rate (1). Although there are many treatments for gastric cancer, it is still a very challenging disease (28). With the development of medical technology, the 5-year survival rate of gastric cancer patients undergoing surgery has steadily increased, but there is still a large proportion of patients with poor prognoses (29). Numerous researches have shown that gastric cancer patients’ prognoses were related to disease tumor markers, body nutrition and immune status (30–32). Therefore, there is a need to develop a more accurate prognostic risk stratification system to stratify patients and help individualize the choice of treatment.

Our study is the first to assess the association between the PNI-IgM score, which a composite indicator of immunity and nutrition, clinicopathological factors and survival. It also demonstrated that the PNI-IgM score predicted prognosis among gastric cancer patients who underwent resection. We found that low PNI and IgM were associated with poor patient prognosis. This study found that age, pTNM stage, radical resection, and CA724 were independent prognostic factors for DFS and OS. In addition, an independent prognostic factor for OS was melaena. In the Stratified analyses, we found age <60 years and CA724 < 2.11 U/m had shorter OS in PNI-IgM score 1.

Although many studies have confirmed the prognostic association of PNI and IgM with gastric cancer (33–35) and other solid tumors (36–38). In this study, PNI and IgM were also demonstrated to predict the prognosis of gastric cancer patients. There are several possible mechanisms explaining the association of PNI-IgM score with the prognosis of patients with gastric cancer. PNI is a composite indicator consisting of albumin and lymphocytes, which mainly reflects the nutritional status of the body, but also the immune status of the body (39, 40). Albumin is a well-known indicator of the nutritional status of the body, and many studies have concluded that low albumin is a sign of malnutrition in the body (41, 42). Lymphocytes are an important component of the body’s immune system and reflect the overall immune status of the body (43). Low serum lymphocytes have been reported to be associated with tumor progression and metastasis in patients with gastric cancer (44–46). IgM accounts for about 10% of the total serum immunoglobulins and mainly reflects the recent immune response. Tumor-reactive IgM had been shown to eliminate malignant cells through complement fixation (47), induction of apoptosis (48), and induction of secondary immune responses against neoantigens (49).

This study also has some limitations. First, this was a single-region, single-center retrospective study with limited sample size and potential selection bias. Second, we only selected patients with gastric cancer who underwent surgery. Third, the cut-off value of PNI and IgM is usually derived from the ROC curve, and the optimal cut-off value is uncertain. Therefore, multi-regional, multi-center, and larger sample size studies are needed to validate our findings.

## Conclusion

In conclusion, our study found that the PNI-IgM score is a valid scoring tool for patients with gastric cancer who received surgery. Patients in the PNI-IgM score 1 group had a worse prognosis than those in the PNI-IgM score 2 group, and the PNI-IgM score 3 group. Therefore, the PNI-IgM score could be used as a biomarker to develop better treatment strategies for patients undergoing surgery for gastric cancer.

## Data availability statement

The raw data supporting the conclusions of this article will be made available by the authors, without undue reservation.

## Ethics statement

This study was approved by the ethics committee of Harbin Medical University Cancer Hospital. All patients provided written informed consent before the study.

## Author contributions

Writing-original draft and Writing-review & editing: ZD, HaS; Data curation and Investigation: RZ, GD, and HP; Methodology and Supervision: YZ, RH; Resources, Funding acquisition and Project administration: YX, and HoS. All authors contributed to the article and approved the submitted version.
